# Supplementary material for: Factors associated with the use of maternal health services by mothers in a post-conflict area of western Côte d’Ivoire in 2016
Source: BMC Health Serv Res. 2020 Feb 22;20:136. doi: 10.1186/s12913-020-4976-2 (PMC7036223; doi:10.1186/s12913-020-4976-2)
Supplement: Supplementary file 1 — Additional file 1. Questionnaire to pregnant women or women who have been pregnant for 3 years prior to survey [file 12913_2020_4976_MOESM1_ESM.pdf]

QUESTIONNAIRE TO PREGNANT WOMEN OR WOMEN WHO HAVE BEEN PREGNANT FOR 3 YEARS PRIOR TO SURVEY

Consent Form

Good morning, madam,

Our study is carried out as part of a Master's thesis in Public Health at the University Félix Houphouët.

The theme of our study is: Factors associated with the use of maternal health services by mothers in the department of Blolequin.

This questionnaire is addressed to you and focuses on understanding the reasons for the use of maternal health services. We hope that the analysis of your answers will help to improve the use of maternal health services by women of childbearing age in the department of Blolequin.

Therefore, we would like to ask you to participate in our study. This questionnaire will take approximately 30 minutes to complete and your answers will remain anonymous. We thank you in advance for your help.

Authorisation

I, the undersigned ………………………………………………………………….., agree to participate in the above-mentioned survey and hereby authorize the use of the data collected about me for the purposes of this study.

Signature of respondent :

Name of surveyor

Date :

Identification of respondent and surveyor

1. Respondent's code ____

2. Location ____

3. Place of the survey

3.1-Residence

3.2-Health center, please, specify name and place :

____

3.3.Other, please specify ____

4. Interview date : |_|_| - |_|_| - |_|_|_|_|

jj m m aaaa

5. Surveyor's name _________________________

A. Household Socioeconomic Characteristics and Maternal Health Practice			
**Q. No**	**Questions & Filters**	**Code**	**Rep.**
A1	What is your birth date? In case the exact year is not known, please give an approximate age	Year of birth ______________<br>1. Age approximate<br>____________________	
A2	Marital status	1. Never married<br>2. Married or in cohabitation<br>3. Divorced or separated<br>4. Widow/ widower<br>99. Refused to respond	
A3	If married or in cohabitation	1. Monogamous<br>2. Polygamist<br>3. 99. Refused to respond	
A4	Residence	1. Urban<br>2. Rural	
A5	Who are you living with ?	1. Husband<br>2. In-laws<br>3. My parents<br>4. By myself<br>5. Others	
A6	How many people live in the household ?	1. Two<br>2. Others	
A7	Who is the decision maker in the household ?	1. Partner<br>2. Wife<br>3. Others	

A. Household Socioeconomic Characteristics and Maternal Health Practice			
**Q. No**	**Questions & Filters**	**Code**	**Rep.**
A8	What kind of work do you do? I mean, what kind of activities keep you busy in average a day, if whether or not you're making money from such activities.<br>Response: ____________________<br>Then Choose the appropriate option:	1. Agriculture<br>2. Housewife<br>3. Craftswoman<br>4. Highschool/ university student<br>5. Civil servant<br>6. Unemployed<br>7. Other	
A9	How much do you earn in CFA F a month ?	1. No earnings<br>2. Less than FCFA 15,000<br>3. Between FCFA 15,000 and FCFA 30,000<br>4. Over FCFA 30,000	
A10	How many pregnancies have you had including abortions ?	1. One<br>2. Other	
A11	Have you ever had to deal with the services of family planning?	1. Yes<br>2. No	
A12	If yes, which one(s) ?	1. Combined oral contraceptives<br>2. Monthly injectables<br>3. Progestational pill<br>4. Implants<br>5. Male and female condoms<br>6. Spermicides<br>7. Female sterilization<br>8. Other	
A13	If no, why ?	1. Lack of information<br>2. Other	

A. Household Socioeconomic Characteristics and Maternal Health Practice			
**Q. No**	**Questions & Filters**	**Code**	**Rep.**
A14	In the past three (3) months, have you been to a Health facility in case of common illnesses ?	1. Yes<br>2. No	
A15	If no, why (many possibles responses) ?	1. Do you use traditional medicines ?<br>2. Other	
A16	If yes, which type ?	1. Plants<br>2. Decoction<br>3. Others	
A17	How much do you spend in average for your traditional care ?	1. FCFA 1,000<br>2. FCFA 2,000<br>3. Other	
A18	Do you use medication under prescription ?	1. Yes<br>2. No	
A19	If no, why ?	1. It's no big deal<br>2. I had no money<br>3. Other	
A20	Did you use an (or some) antenatal consultation (s) during this regnancy ?	1. Yes<br>2. No	
A21	If yes, how many antenatal consultations have you had ?	1. Less than four<br>2. Equal or more than four	
A22	Who did you meet during your consultations?	1. Modern doctor<br>2. Traditional doctor	
A23	How much did you pay during your consultations (average cost / CPN) cash ?	1. FCFA 1,000<br>2. FCFA 2,000<br>3. Other	

A. Household Socioeconomic Characteristics and Maternal Health Practice			
**Q. No**	**Questions & Filters**	**Code**	**Rep.**
A24	Were yoy satisfied with the service ?	1. Yes<br>2. No	
A25	If no**,** say why ?	1. Bad reception<br>2. Reception time too long<br>3. Other	
A26	Did the health agent work out a pregnancy plan with you?	1. Yes<br>2. No	
A27	Did you have an assisted delivery in a health facility during your pregnancy?	1. Yes<br>2. No	
A28	If no, say why ?	1. No qualified staff<br>2. Other	
A29	How much did your last pregnancy facility ?	1. Free of charge<br>2. Other	
A30	Did you use a post-natal consultation aft delivery ?	1. Yes<br>2. No	
A31	If no, say why ?	1. No money<br>2. Other	
A32	Are you cultures and customes likely to affect the attendance of health facilities during pregnancy ?	1. Yes<br>2. No	
A33	If yes, how ?	1. Penalty<br>2. Other	
A34	Is your pregnancy desired ?	1. Yes<br>2. No	

B. Constraints related to access to maternal health services			
**Q. No**	**Questions & Filters**	**Code**	**Rep.**
B1	If you did not have access to health facilities It's just because of one of the following reasons	1. You don't have enough money<br>2. The health facility is remote<br>3. the prescriptions are long and expensive<br>4. the care is effective<br>5. Reception is poor<br>6. There are no female staffs<br>7. No insurance coverage or health mutual<br>8. Other	
B2	Who pays for the health care for women ?	1. Own income<br>2. Partner<br>3. The healyh mutual<br>4. Parents<br>5. Private insurance<br>6. The state<br>7. Other	
B3	How much did the the care and support cost In average during your last pregnancy ?<br>(in CFA F)	1. I don't know<br>2. Other	
B4	How much did the average transportation cost during your last pregnancy ?	1. I don't know<br>2. Other	

B. Constraints related to access to maternal health services			
Q. No	Questions & Filters	Code	Rep.
B5	How much do you think the monthly expenses for your household are in FCFA ?	1. from 0 to 30,000<br>2. from 30,000 to 60,000<br>3. from 60,000 to 100,000<br>4. from 100,000 to 150,000<br>5. from 150,000 to 300,000<br>6. over 300,000	
B6	Did you test for HIV /AIDS during your pregnancy ?	1. Yes<br>2. No	
B7	If no, why	1. I don't know<br>2. Other	
B8	How far is the facility from your house ?	1. less than 5 kms<br>2. Equal to 5 kms<br>3. Over 5 kms	

B. Constraints related to access to maternal health services			
**Q. No**	**Questions & Filters**	**Code**	**Rep.**
B5	How much do you think the monthly expenses for your household are in FCFA ?	1. from 0 to 30,000<br>2. from 30,000 to 60,000<br>3. from 60,000 to 100,000<br>4. from 100,000 to 150,000<br>5. from 150,000 to 300,000<br>6. over 300,000	
B6	Did you test for HIV /AIDS during your pregnancy ?	3. Yes<br>4. No	
B7	If no, why	3. I don't know<br>4. Other	
B8	How far is the facility from your house ?	4. less than 5 kms<br>5. Equal to 5 kms<br>6. Over 5 kms	

Thank you for your participation
